# Supplementary material for: Extraction Solvents Affect Anthocyanin Yield, Color, and Profile of Strawberries
Source: Plants (Basel). 2023 Apr 29;12(9):1833. doi: 10.3390/plants12091833 (PMC10181068; doi:10.3390/plants12091833)
Supplement: Supplementary file 1 [file plants-12-01833-s001.zip › plants-2356788-supplementary.pdf]

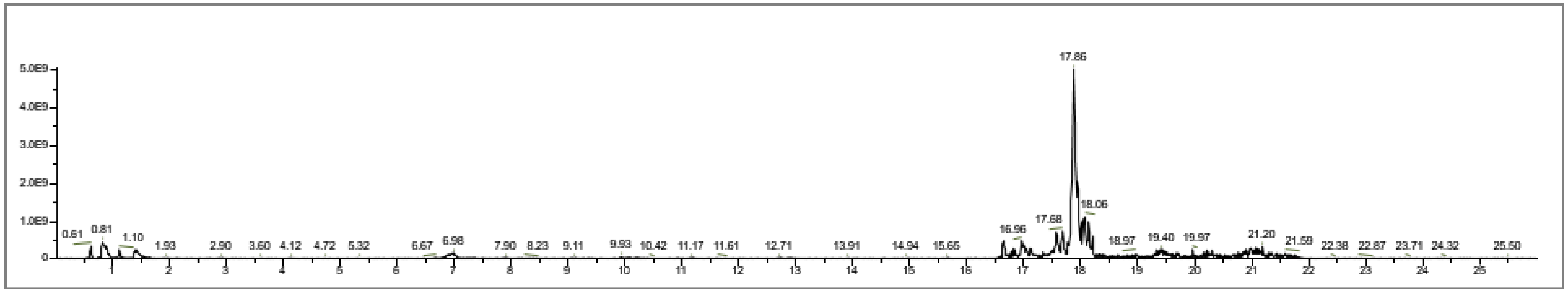

A: Chloroform: methanol (Solovchenko et al. [6])

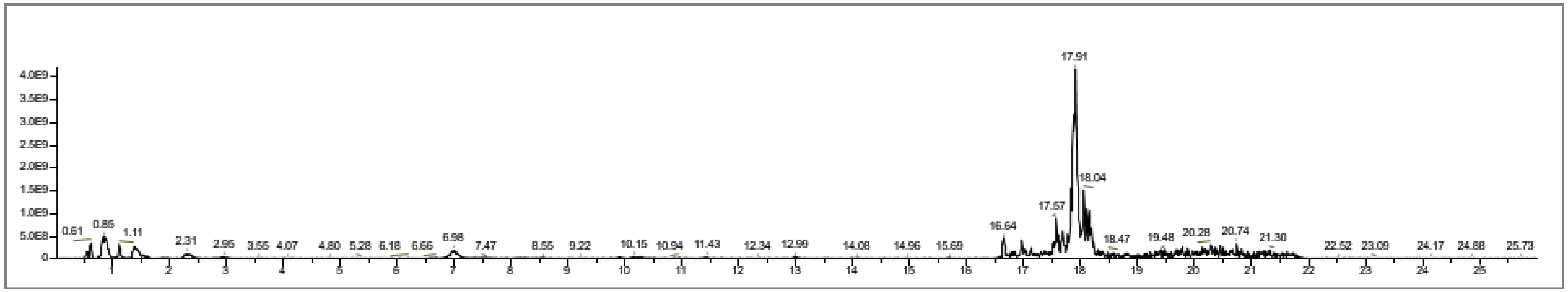

B: Methanol (Solovchenko et al. [6])

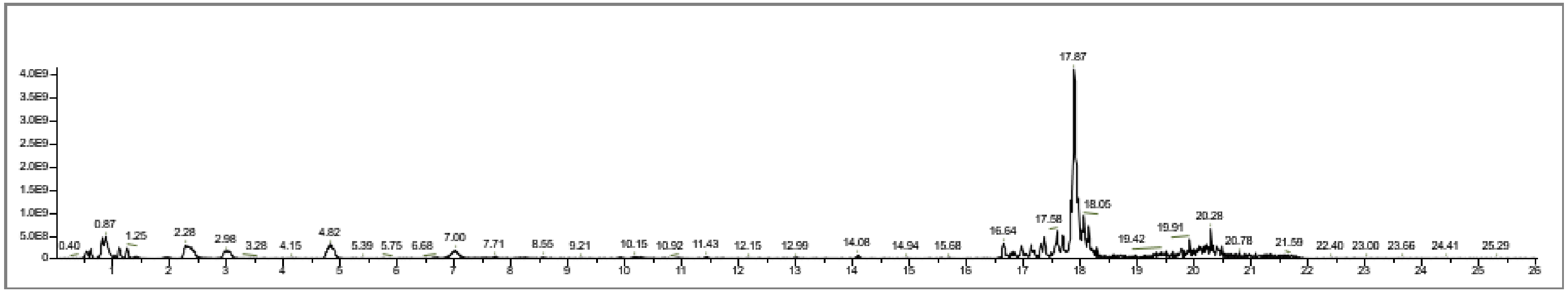

C: Methanol: water (Lindoo and Caldwell [7])

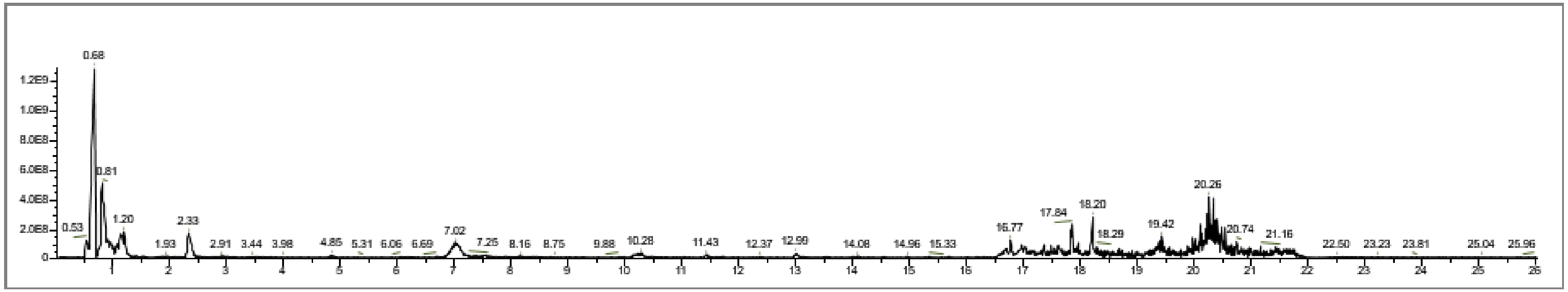

D: pH differential, pH 1.0 (Lee et al. [11])

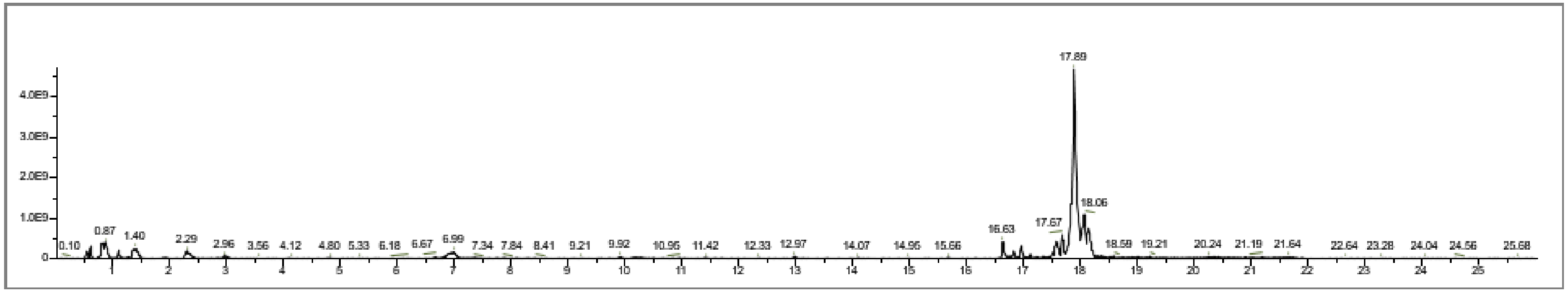

E: Methanol: water (Neff and Chory [9])

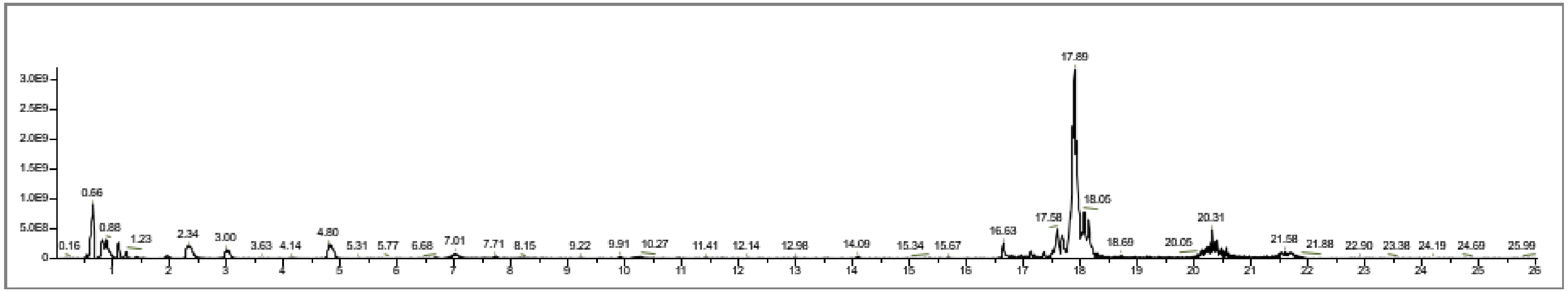

F: Combined solvents (Gauch et al. [16])

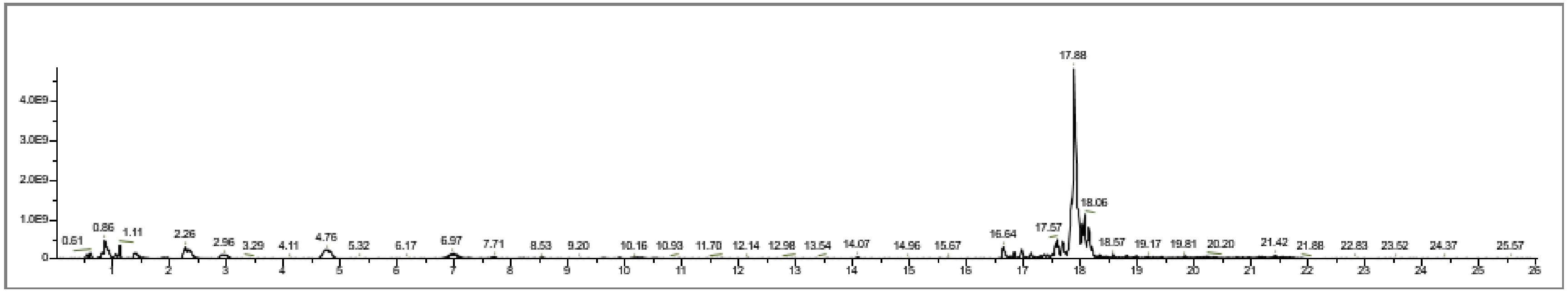

G: Ethanol (Karaaslan and Yaman [12])

Supplementary Figure S1: The mass spectra of anthocyanidins/anthocyanins extracted by different solvents from freshly-pureed strawberries and identified by ultra-high liquid chromatography-electrospray ionization tandem mass spectrometry (UHPLC-ESI-MS) with retention times identified.
Notes: The solvents were A) Chloroform:methanol (Solovchenko et al. [6]), B)methanol (Solovchenko et al. [6]), C) methanol: water (Lindoo and Caldwell [7]), D) pH differential buffers (Lee et al. [11]), E) methanol: water (Neff and Chory [9]), F) combined solvents (methanol: water followed by pH differential; Gauch et al. [16]), G) ethanol (Karaaslan & Yaman [12]). The solvents were acidi-fied by HCl (0.1%), except in Lindoo and Caldwell (1% HCl) and pH differential and com-bined solvents, where HCl was added to reach a certain pH (1.0 and 4.5). The extracts were stored at 4 °C for 48h before analyzing for anthocyanin profile.
